# Mid-infrared spectra of dried and roasted cocoa (*Theobroma cacao* L.): A dataset for machine learning-based classification of cocoa varieties and prediction of theobromine and caffeine content

**DOI:** 10.1016/j.dib.2024.111243

**Published:** 2024-12-19

**Authors:** Gentil A. Collazos-Escobar, Andrés F. Bahamón-Monje, Nelson Gutiérrez-Guzmán

**Affiliations:** a Centro Surcolombiano de Investigación en Café (CESURCAFÉ), Departamento de Ingeniería Agrícola, Universidad Surcolombiana, Neiva-Huila 410001, Colombia; b Grupo de Análisis y Simulación de Procesos Agroalimentarios (ASPA), Instituto Universitario de Ingeniería de Alimentos–FoodUPV, Universitat Politècnica de València, Camí de Vera s/n, Edificio 3F, València 46022, España

**Keywords:** Functional groups, Cocoa quality monitoring, Multivariate statistical tools, Artificial intelligence, Real-time decision-making

## Abstract

This paper presents a comprehensive dataset of mid-infrared spectra for dried and roasted cocoa beans (*Theobroma cacao* L.), along with their corresponding theobromine and caffeine content. Infrared data were acquired using Attenuated Total Reflectance-Fourier Transform Infrared (ATR-FTIR) spectroscopy, while High-Performance Liquid Chromatography (HPLC) was employed to accurately quantify theobromine and caffeine in the dried cocoa beans. The theobromine/caffeine relationship served as a robust chemical marker for distinguishing between different cocoa varieties. This dataset provides a basis for further research, enabling the integration of mid-infrared spectral data with HPLC (as a standard) to fine-tune machine learning and deep learning models that could be used to simultaneously predict the theobromine and caffeine content, as well as cocoa variety in both dried and roasted cocoa samples using a non-destructive approach based on spectral data. The tools developed from this dataset could significantly advance automated processes in the cocoa industry and support decision-making on an industrial scale, facilitating real-time quality control of cocoa-based products, improving cocoa variety classification, and optimizing bean selection, blending strategies, and product formulation, while reducing the need for labor-intensive and costly quantification methods. The dataset is organized into Excel sheets and structured according to experimental conditions and replicates, providing a valuable framework for further analysis, model development, and calibration of multivariate statistical models.

Specifications Table

**Table:** 

Subject	Food engineering
Specific subject area	Food technology, Food engineering, Food Science
Type of data	Excel files (Origin of cocoa samples, initial characterization of dried and roasted cocoa samples, theobromine and caffeine content of dried cocoa samples, mid-infrared spectra of dried cocoa samples and mid-infrared spectra of roasted cocoa samples). Figure (Mid-infrared spectra of dried and roasted cocoa and experimental procedure for dataset acquisition).
Data collection	Mid-infrared spectra (Attenuated Total Reflectance-Fourier Transform Infrared, ATR-FTIR) and Theobromine and caffeine content (High-Performance Liquid Chromatography, HPLC).
Data source location	The experimental dataset described in this study has been obtained in the Centro Surcolombiano de Investigación en Café (CESURCAFÉ) from the Universidad Surcolombiana, Neiva-Huila, Colombia.
Data accessibility	Repository name: Mendeley Data Data identification number: 10.17632/wct3y9t8cs.1 Direct URL to data: https://data.mendeley.com/datasets/wct3y9t8cs/1
Related research article	G. A. Collazos-Escobar, Y. F. Barrios-Rodriguez, A. F. Bahamón-Monje, N. Gutiérrez-Guzmán, Mid-infrared spectroscopy and machine learning as a complementary tool for sensory quality assessment of roasted cocoa-based products, Infrared Physics and Technology. 141 (2024) 105,482.

## 1 Value of the Data

• This dataset offers a highly valuable tool for the classification of cocoa varieties. By establishing a correlation between mid-infrared spectral data and methylxanthine content (theobromine/caffeine) it enables precise, non-destructive identification of cocoa genotype. This is crucial for maintaining quality control in cocoa-based products. Given the inherent variability among cocoa cultivars, the ability to accurately classify varieties and optimize their processing is a significant challenge in the cocoa industry. Employing this dataset could significantly aid the industry in overcoming this challenge.
• This dataset provides a framework for building predictive models. Mid-infrared spectra combined with theobromine and caffeine content can be integrated with machine learning and multivariate statistical methods to develop a robust model that accurately predicts theobromine and caffeine content based on a non-destructive and rapid technique such as mid-infrared spectroscopy. These models could also be applied for real-time quality inspection of cocoa-based products and to support real-time decision-making in the cocoa industry.
• This dataset provides valuable insights for cocoa producers, researchers, food scientists, and industry professionals by serving as a critical tool for calibrating robust mathematical models that support decision-making. It offers significant benefits for real-time quality control in the cocoa industry, enabling the development of classifiers for cocoa variety identification and predictive models for chemical composition. These models are crucial for optimizing processes such as bean selection, blending, and product formulation. By reducing dependence on time-consuming techniques including HPLC, this dataset helps drive the industry toward Food Quality 4.0, promoting greater efficiency and process improvements.
• The dataset on mid-infrared spectra of dried and roasted cocoa has various applications, including the identification of functional groups related to the chemical composition of the food product, such as lipids, proteins, and polyphenols. Additionally, this technique can be used to detect quality issues, identify adulterants, and observe changes occurring during post-harvest processing. It allows for the characterization of chemical transformations, the quantification of bioactive compounds, including antioxidants, the determination of origin, and the detection of contaminants or adulterations. This approach is crucial for both scientific research and process optimization in the food industry.

## 2 Background

The cocoa industry faces significant challenges due to the high genetic variability of cocoa cultivars. This variability complicates the management of quality and consistency across cocoa batches from diverse growing regions, negatively impacting both product quality and economic viability. The genotypic variation in cocoa beans is closely associated with the theobromine/caffeine ratio (methylxanthine content), which serves as a crucial marker for differentiating cocoa varieties based on their chemical profiles. Traditional classification methods for determining cocoa variety, particularly those based on High-Performance Liquid Chromatography (HPLC), are resource-intensive and time-consuming, requiring considerable amounts of chemical reagents, specialized equipment, and trained personnel. In response to these challenges, Attenuated Total Reflectance-Fourier Transform Infrared (ATR-FTIR) spectroscopy emerges as a promising, non-destructive, and cost-effective alternative for classifying cocoa varieties while ensuring quality control. This rapid and economically feasible technique enables the development of robust statistical models by correlating spectral data with standard HPLC results, thus facilitating accurate classification of cocoa products. Moreover, mid-infrared spectral data can also be utilized to calibrate multivariate models capable of simultaneously predicting theobromine and caffeine content in cocoa beans. This advancement significantly enhances quality control measures within both the food and pharmaceutical industries. Furthermore, this integration provides a robust framework for addressing the genetic variability of cocoa cultivars, offering automated solutions for variety classification and simultaneous prediction of theobromine and caffeine content. This approach mitigates the dependence on traditional, labour-intensive methods such as HPLC, thereby improving efficiency in managing cocoa quality and ensuring product consistency.

## 3 Data Description

The experimental dataset was compiled into five Excel files, each described in detail below. These files include:

**Sample_origin:** This Excel file contains information about the location and origin of fresh cocoa pod samples. The first column corresponds to the cultivar number, while the second to fourth columns provide details on the municipality, village name, and the farm where the samples were collected. Additionally, the last three columns include the geographical coordinates and altitude of these cultivars. This information is valuable for the geographical analysis of cocoa production, as it facilitates the clustering of growing areas based on their chemical and spectral characteristics.

**Initial_characterization:** This Excel file contains the initial characterization data for dried and roasted cocoa samples. The first sheet presents the moisture content and water activity of dried cocoa samples, while the second sheet provides the initial moisture content and water activity of roasted cocoa samples (the method to determine them is detailed in the EXPERIMENTAL DESIGN, MATERIALS AND METHODS section). In each sheet, the first column indicates the sample number, and the determination of moisture content and water activity was performed in triplicate for each sample. These initial parameters provide a standardized baseline for further analysis, allowing for the consideration of water content in the mathematical modeling.

**Theobromine_Caffeine:** This Excel file contains the quantification of theobromine and caffeine in dried cocoa samples, measured by HPLC (as detailed in the EXPERIMENTAL DESIGN, MATERIALS, AND METHODS section). The first two columns contain the sample identifiers and replicates (triplicate), while the third and fourth columns provide the theobromine and caffeine values in mg of theobromine/caffeine per gram of cocoa sample on a dry basis. The fifth and sixth columns compute the relationships between theobromine and caffeine content and cocoa genotype, respectively. The cocoa variety was defined as follows: a theobromine/caffeine ratio greater than 9 for the Forastero genotype and a ratio between 3 and 9 for the Trinitario genotype [1]. The theobromine and caffeine content, as well as cocoa genotype, can be used as response variables in the calibration of predictive and classification statistical models.

**Mid_InfraredSpectra_driedCocoa:** This Excel file presents the mid-infrared spectra of dried cocoa samples. The mid-infrared spectra were acquired using the ATR-FTIR technique (as detailed in the EXPERIMENTAL DESIGN, MATERIALS, AND METHODS section). The first column contains the wavenumber (cm⁻¹) for the entire infrared spectrum, while every three columns from columns 2 to 165 contain the spectra of each sample in triplicate. A total of 55 samples were recorded in triplicate, resulting in 165 spectra. Furthermore, the last column (column 166) presents the average spectrum of the 165 samples. These spectra, collected from dried cocoa beans, provide valuable insights into the chemical composition and functional groups of the samples. They can also be used as inputs to calibrate multivariate statistical models, such as machine learning and deep learning, to classify cocoa varieties based on the defined theobromine/caffeine relationship and to predict both theobromine and caffeine content.

**Mid_InfraredSpectra_roastedCocoa:** This Excel file presents the mid-infrared spectra of roasted cocoa samples. The mid-infrared spectra were acquired using the ATR-FTIR technique (as detailed in the EXPERIMENTAL DESIGN, MATERIALS, AND METHODS section). The first column contains the wavenumber (cm⁻¹) for the entire infrared spectrum, while every three columns from columns 2 to 165 contain the spectra of each sample in triplicate. A total of 55 samples were recorded in triplicate, resulting in 165 spectra. Furthermore, the last column (column 166) presents the average spectrum of the 165 samples. These spectra, collected from roasted cocoa beans, provide valuable insights into the chemical composition and functional groups of the samples. They can also be used as inputs to calibrate multivariate statistical models, such as machine learning and deep learning, to classify cocoa varieties based on the defined theobromine/caffeine relationship.

**Fig. 1:**
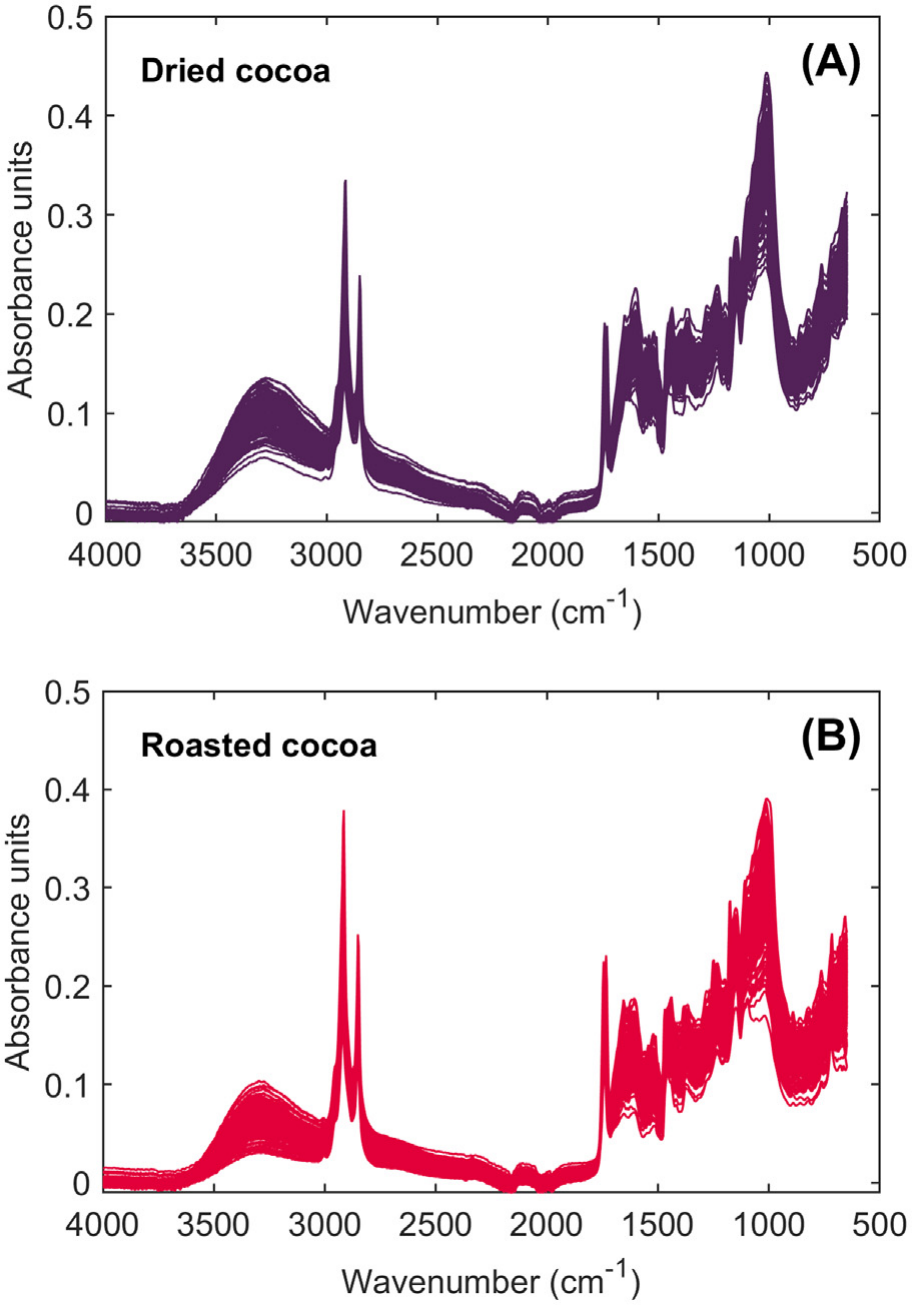
Raw mid-infrared spectra of dried (A) and roasted (B) cocoa samples recorded in the wavenumber range of 4000–500 cm^–1^.

In Fig. 1, the raw mid-infrared spectra of dried (Fig. 1A) and roasted cocoa (Fig. 1B) samples are illustrated.

## 4 Experimental Design, Materials and Methods

**Fig. 2:**
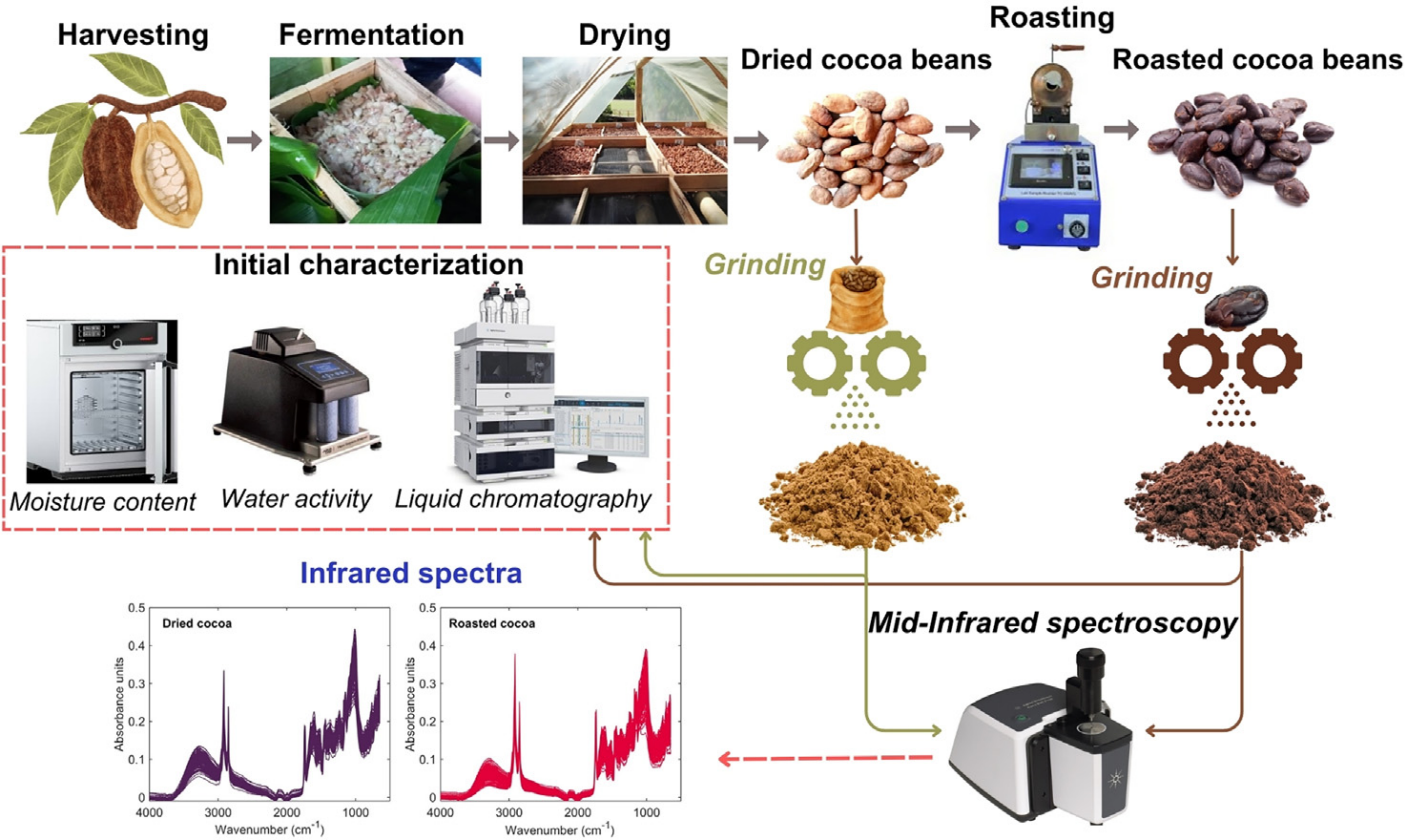
Flow chart illustrating the experimental procedure used to determine mid-infrared spectra and theobromine/caffeine content of cocoa samples for obtaining the dataset.

The processing procedure illustrating the experimental methodology applied to process the dried and roasted cocoa samples for obtaining the dataset is described in Fig. 2. Fifty-five cocoa pod samples (*Theobroma cacao* L., 60 kg each) were collected from different cocoa-growing areas in the Huila region of Colombia (detailed in **Sample_origin**). Once the samples were collected, they were stored in jute bags and transported for 3 h to be processed at the Centro Surcolombiano de Investigación en Café (CESURCAFÉ) in Neiva-Huila, Colombia.

Firstly, the cocoa beans were manually separated from the pods and immediately fermented in wooden boxes for seven days (Fig. 2). During the fermentation process, the temperature was monitored every 5 h. In the first 72 h of cocoa fermentation, the cocoa mass reached 45 °C, during which the anaerobic phase promoted the death of the germ. Subsequently, fermentation proceeded in an aerobic phase, with cocoa mass temperatures ranging between 25 and 28 °C. The samples were then sun-dried (Fig. 2) until they reached a moisture content of 6–7 % on a wet basis (w.b.). During the drying process, the moisture content of the beans was monitored with a grain moisture tester (G600, Gehaka AGRA, Brazil). After drying, only healthy cocoa beans (without insect damage or being over/under-fermented) were selected for analysis [2].

For each dried cocoa sample, 150 g were roasted using rotary equipment (TC-150R, Quantik, Colombia), which allowed for precise control of time and temperature. The roasting process was conducted at 120 ± 2 °C for 27 min. After this process, the beans were manually dehulled to obtain the roasted cocoa samples. Both dried and roasted cocoa beans were independently ground using a rotary knife grinder (FPSTFP3322, Oster®, Colombia) to obtain ground samples (Fig. 2). These samples were initially characterized by measuring moisture content and water activity. For moisture content determination, 10 g of dried and roasted cocoa samples were dried in an oven (UF55, Memmert GmbH+Co. KG, Germany) at 105 °C for 24 h until reaching constant weight. Results were gravimetrically expressed in wet basis. The water activity of cocoa samples was determined in triplicate using a vapor sorption analyzer (VSA Aqualab, Decagon Devices, Inc. Pullman, WA) [3].

Theobromine and caffeine contents were determined using an HPLC instrument (Agilent 1260 Infinity II series liquid chromatography, Agilent Technologies, Santa Clara, CA, USA) with a Poroshell 120-C18 (2.7 μm, 4.6 × 150 mm) column. The reversed-phase process was performed with an injection volume of 20 μL and a flow rate of 1 mL/min. Analyte separation was carried out using isocratic elution with methanol (Merck, Darmstadt, Germany) and water containing 0.2 % acetic acid (20:80 v/v) for 10 min. The detection process of these analytes was performed using a diode array detector (DAD) at 280 nm. Both theobromine and caffeine were identified by comparing their retention times and ultraviolet visible spectra with their corresponding chemical standards [4].

Mid-infrared spectra of dried and roasted cocoa samples were obtained using a Fourier-transform infrared (FTIR) spectrophotometer (Cary 630, Agilent Technologies, USA) equipped with a horizontal Attenuated Total Reflectance (ATR) accessory featuring a diamond ATR and a ZnSe cell. The ATR-FTIR analysis was conducted in a controlled environment at 25 ± 0.5 °C [2]. Approximately 1 g of ground dried and roasted cocoa samples were placed in the ATR accessory and compressed. Background readings were taken prior to each sample measurement. Spectral data were recorded in the wavenumber range of 4000–650 cm^–1^, with a resolution of 4 cm^–1^ and a scan rate of 16 [5]. The raw mid-infrared spectra were compiled in both **Mid_InfraredSpectra_driedCocoa** and **Mid_InfraredSpectra_roastedCocoa.** These spectra were reported as raw signals to be pre-processed using baseline correction, standard normal variate (SNV), multiplicative scatter correction (MSC), first derivative (1D), and second derivative (2D) depending on the application and with the aim of assessing the influence of spectral pre-processing on statistical results in both classification and regression models.

## Limitations

This study represents an initial step toward the development of a comprehensive dataset to support the advancement of machine learning and deep learning models for classifying cocoa genotypes and predicting the theobromine and caffeine content in dried and roasted cocoa beans from different growing areas in Huila-region of Colombia. Future research should include samples from diverse geographical origins and climatic conditions, while also incorporating variables such as agricultural practices, fermentation processes, drying and roasting parameters into the mathematical modeling process. Incorporating these factors could significantly enhance the robustness of predictive and classification models by accounting for the complex variability inherent in cocoa production systems. These improvements would further enhance the accuracy and effectiveness of non-destructive mid-infrared spectroscopy techniques for prediction and classification tasks. Additionally, future research should focus on integrating genetic data into machine learning models, as potential genetic mutations driven by geographical and environmental factors may affect the precision of classification and prediction techniques. By expanding the dataset to include samples from diverse production regions and accounting for agronomic, edaphoclimatic, and genetic variability, multivariate modeling approaches can better address the inherent complexity of cocoa, thereby reducing uncertainty and yielding more reliable and generalizable results.

## Ethics Statement

The dataset acquired in this study did not involve human subjects, animal experiments, or data obtained from social media platforms.

## CRediT authorship contribution statement

**Gentil A. Collazos-Escobar:** Conceptualization, Methodology, Software, Data curation, Visualization, Writing – original draft. **Andrés F. Bahamón-Monje:** Software, Data curation, Writing – original draft. **Nelson Gutiérrez-Guzmán:** Supervision, Writing – review & editing.

## Data Availability

Mendeley DataInfrared spectral data of dried and roasted cocoa (Theobroma cacao L.) for calibrating classification models of cocoa varieties and predicting theobromine and caffeine content (Original data)

## References

[1] Collazos-Escobar G.A., Barrios-Rodriguez Y.F., Bahamón-Monje A.F., Gutiérrez-Guzmán N. (2023). Uses of mid-infrared spectroscopy and chemometric models for differentiating between dried cocoa bean varieties. Rev. Brasil. Engenharia Agric. Ambient..

[2] Collazos-Escobar G.A., Barrios-Rodríguez Y.F., Bahamón-Monje A.F., Gutiérrez-Guzmán N. (2024). Mid-infrared spectroscopy and machine learning as a complementary tool for sensory quality assessment of roasted cocoa-based products. Infrared Phys. Technol..

[3] Collazos-Escobar G.A., Gutiérrez-Guzmán N., Váquiro-Herrera H.A., Bon J., Garcia-Perez J.V. (2022). Thermodynamic analysis and modeling of water vapor adsorption isotherms of roasted specialty coffee (Coffee arabica L. cv. Colombia). LWT.

[4] Batista N.N., de Andrade D.P., Ramos C.L., Dias D.R., Schwan R.F. (2016). Antioxidant capacity of cocoa beans and chocolate assessed by FTIR. Food Res. Int..

[5] Collazos-Escobar G.A., Gutiérrez-Guzmán N., Váquiro-Herrera H.A., Amorocho-Cruz C.M. (2020). Water dynamics adsorption properties of dried and roasted cocoa beans (theobroma cacao L.). Int. J. Food Prop..

